# Towards a revision of the South American genus *Praocis* Eschscholtz (Coleoptera, Tenebrionidae), with estimation of the diversity of each subgenus

**DOI:** 10.3897/zookeys.415.6656

**Published:** 2014-06-12

**Authors:** Gustavo E. Flores, Jaime Pizarro-Araya

**Affiliations:** 1 CONICET, Laboratorio de Entomología, Instituto Argentino de Investigaciones de las Zonas Áridas (IADIZA, CCT CONICET Mendoza), Casilla de correo 507, 5500 Mendoza, Argentina; 2 Laboratorio de Entomología Ecológica, Departamento de Biología, Facultad de Ciencias, Universidad de La Serena, Casilla 599, La Serena, Chile

**Keywords:** Taxonomy, Pimeliinae, Praociini, *Praocis*, key, diversity, South America

## Abstract

A review of the subgenera of the South American genus *Praocis* Eschscholtz (Pimeliinae: Praociini) is presented. *Praocis* comprises 77 species and 8 subspecies arranged in nine subgenera distributed in arid lands from Central Peru and Bolivia to the Southern part of Patagonia in Chile and Argentina. For each subgenus of *Praocis*: *Praocis* Eschscholtz, *Mesopraocis* Flores & Pizarro-Araya, **subgen. n.**, *Anthrasomus* Guérin-Méneville, *Filotarsus* Gay & Solier, *Postpraocis* Flores & Pizarro-Araya, **subgen. n.**, *Hemipraocis* Flores & Pizarro-Araya, **subgen. n.**, *Orthogonoderes* Gay & Solier, *Praonoda* Flores & Pizarro-Araya, **subgen. n.**, and *Praocida* Flores & Pizarro-Araya, **subgen. n.**, we present a diagnosis using new and constant characters of adult morphology such as clypeal configuration, length and proportion of antennomeres 9, 10 and 11, arrangement of apical tomentose sensory patches on antennomeres 10 and 11, anterior margin of prosternum, lateral margin of elytron, ventral surface of profemora, and shape of protibiae. An identification key for the nine subgenera of *Praocis* is presented. Type species are designated for the five new subgenera; for *Mesopraocis*: *Praocis calderana* Kulzer, for *Postpraocis*: *Praocis pentachorda* Burmeister, for *Hemipraocis*: *Praocis sellata* Berg, for *Praonoda*: *Praocis bicarinata* Burmeister, for *Praocida*: *Praocis zischkai* Kulzer, and for the previously described subgenus *Orthogonoderes*: *Praocis subreticulata* Gay & Solier. The current number of species and the estimated number of species to be described are presented. The distribution ranges of the subgenera, including new records from collections and recent expeditions, are given. Habitat preferences and a discussion of the biogeography of the genus are also presented.

## Introduction

**Figures 1–2.**
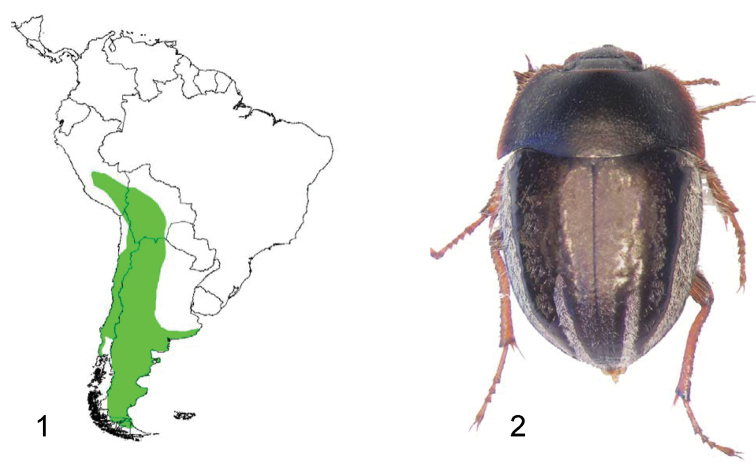
**1** Distribution area of the whole genus *Praocis* **2** Dorsal view of *Praocis (Praocis) bicentenario*, holotype (previously published in Flores and Pizarro-Araya 2012, Zootaxa 3336: Fig. 17; copyright Magnolia Press, reproduced with permission).

The genus *Praocis* Eschscholtz belongs to the Praociini, an endemic Neotropical tribe of Pimeliinae with 151 species arranged in 15 genera (Flores and Pizarro-Araya 2012). *Praocis* is the most specious genus of the tribe (52% of the species). It comprises 77 species and 8 subspecies, arranged in nine subgenera (Flores and Pizarro-Araya 2012), distributed from central Peru to the southern part of Patagonia in Argentina and Chile. The distribution of *Praocis* species coincides with the whole distribution of the tribe (Fig. 1) and is related to the arrangement of the Andes mountain range in arid and semiarid lands of southern South America (Flores and Pizarro-Araya 2006).

The last revision of *Praocis* was made by Kulzer (1958) in the context of a tribal review. Kulzer (1958) classified the species of *Praocis* into 10 subgenera, six of which were new: *Mesopraocis*, *Postpraocis*, *Parapraocis*, *Hemipraocis*, *Praonoda*, and *Praocida*, plus the four previously recognized as valid by Solier (1840): *Praocis* s. str., *Anthrasomus* Guérin-Méneville 1834, *Orthogonoderes* Gay & Solier, 1840, and *Filotarsus* Gay & Solier, 1840. Kulzer (1958) did not characterize his new subgenera nor designate type species, but in his key he mentioned character states for identifying some of them except between *Anthrasomus* and *Filotarsus*, and between *Orthogonoderes* and *Praocida*, which can be keyed only by body size. Kulzer (1958) also failed to assess the geographic distribution of the subgenera, reporting only isolated localities of the species.

The subgeneric classification of the genus was recently reviewed (Flores and Pizarro-Araya 2012) and the genus was redefined on the basis of five constant character states. The subgenus *Parapraocis* was excluded from *Praocis* because its species exhibit different character states from those defining the genus and it was recognized as a separate genus within Praociini (Flores and Pizarro-Araya 2012).

In the current study we report new constant characters to define each *Praocis* subgenus, such as shape of clypeus, frons and clypeal suture, length and proportion of antennomeres 9, 10 and 11, arrangement of apical tomentose sensory patches on antennomeres 10 and 11, and ventral surface of profemora. We also used the characters defined by Kulzer: shape of anterior margin of prosternum, posterior angles of pronotum, lateral margin of pronotum, lateral margin of elytron, shape of body and apical process of protibiae.

The objectives of this study are to present elements for a revision of the genus *Praocis* by incorporating new constant characters from external morphology to define each subgenus, to designate type species for some subgenera that remain unavailable, to estimate the diversity of each subgenus, to detail the geographic distribution and habitat of each subgenus and to report new distributional records for some subgenera.

## Material and methods

**Material examined.** The present study is based on an examination of specimens deposited in the following collections (we follow Arnett et al. 1993 where possible for collection abbreviations):

FMNH Field Museum of Natural History, Chicago, USA

IADIZA Instituto Argentino de Investigaciones de las Zonas Áridas, Mendoza, Argentina

LEULS Laboratorio de Entomología Ecológica, Universidad de La Serena, Chile

MACN Museo Argentino de Ciencias Naturales Bernardino Rivadavia, Buenos Aires, Argentina

MLPA Museo de La Plata, Buenos Aires, Argentina

MNHN Muséum National d’Histoire Naturelle, Paris, France

MNHUB Museum fur Naturkunde der Humboldt Universitat, Berlin, Germany

MNNC Museo Nacional de Historia Natural, Santiago, Chile

NHMB Natural History Museum, Basel, Switzerland

**Type species.** For the subgenera *Praocis*, *Anthrasomus*, and *Filotarsus* the type species were designated prior to this study. For *Orthogonoderes* Gay & Solier (in Solier 1840) the authors characterized this new taxon but did not indicate the type species. We designate the type species of *Orthogonoderes* in this paper (Article 67.4 ICZN 1999) based on one of the six specific names available in the original publication (Article 67.2.1 ICZN 1999).

The remaining five names of the subgenera proposed by Kulzer (1958): *Mesopraocis*, *Postpraocis*, *Hemipraocis*, *Praonoda*, and *Praocida* are unavailable because Kulzer (1958) did not designate type species for these subgenera. To be available, every new genus-group name published after 1930 must, in addition to satisfying the provisions of Article 13.1 (ICZN 1999), be accompanied by the fixation of a type species in the original publication (Article 67.4.1 ICZN 1999). These five names will be made available for the first time in this article. To fix the current interpretation of these names and to ensure stability as these names were used in previous works (Peña 1966; Flores 2007, 2009; Alfaro et al. 2009; Flores et al. 2011; Flores and Pizarro-Araya 2012; Cortés-Contreras et al. 2013), we use the same names proposed by Kulzer (1958), present a diagnosis of each subgenus and hereby designate the type species on the basis of the specific names available for this nomenclatural act, the type specimens are not lost and the species is representative of the characters of the subgenus.

**Figures 3–6.**
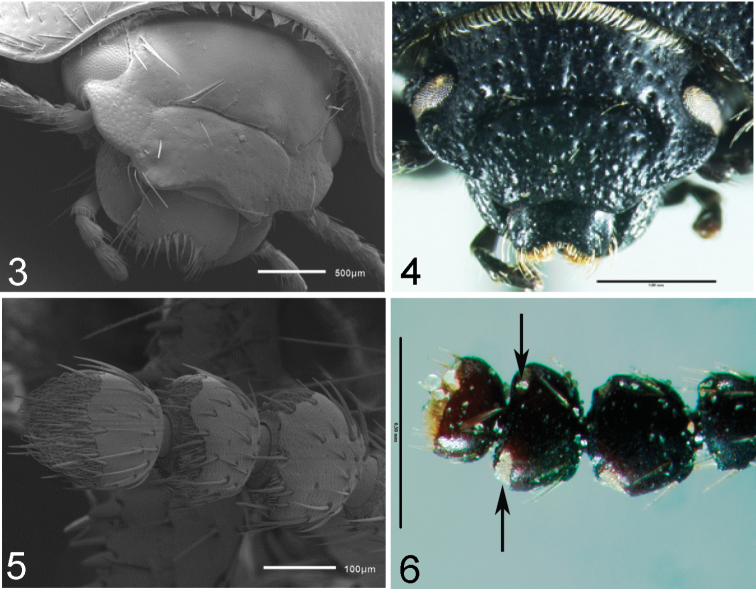
**3** *Praocis (Praocis) subaenea*, head in dorsal view **4** *Praocis (Orthogonoderes) rotundata*, head in frontal view **5** *Praocis (Praocis) bicentenario*, antennomeres 9–11 in dorsal view **6** *Praocis (Orthogonoderes) rotundata*, antennomeres 9–11 in dorsal view (Figs **3** and **5** scanning electron micrographs previously published in Flores and Pizarro-Araya 2012, Zootaxa 3336: Figs **1** and **2**; copyright Magnolia Press, reproduced with permission).

**Characters.** For each subgenus of *Praocis* we present a diagnosis using the following characters and character states:

Clypeal configuration (characters 1–3). The anterior margin of clypeus, in most subgenera, extends beyond the lateral expansion of frons (Fig. 3); in some species of *Filotarsus* it appears at same level as lateral expansion of frons. The width of the anterior margin of the clypeus, in most subgenera, does not exceed half the interocular width (Fig. 3), while in some species of *Filotarsus* it is equal to interocular width. The clypeal suture shows two different states: as horizontal groove (Fig. 4), the clypeus being lower than frons; and as vertical groove, in this state the clypeus and frons are at the same level (Fig. 3).

Length and proportion of antennomeres 9, 10 and 11 (characters 4–5) are very variable among subgenera. Antennomere 9 can be longer than antennomere 10 (Fig. 5) or equal in length to antennomere 10. Antennomere 11 is in most subgenera longer than antennomere 10 (Fig. 5), in *Orthogonoderes* it is shorter than antennomere 10 (Fig. 6) and equal in length to antennomere 10 in *Mesopraocis*.

Arrangement of apical tomentose sensory patches on antennomeres 10 and 11 (characters 6–7) are also variable among the subgenera. The apical tomentose sensory patches on antennomere 10 are arranged in two areas subequal in size (Fig. 6), or in a dorsally continuous semicircle (Fig. 5). On antennomere 11, the apical tomentose sensory patches are located in a single area on the distal third (Fig. 6) or on the distal half of its surface (Fig. 5).

The anterior margin of the prosternum (character 8) presents two states: with a narrow sharp edge or lacking that edge. The lateral margin of the elytron (character 9) can be not defined, rounded, continuous between dorsal area of elytron and pseudopleuron, or well defined by a narrow, sharp carina-shaped edge or by a wide longitudinal, prominent edge. The ventral surface of the profemora (character 10) presents a row of setae on the anterior edge or lacks that row of setae. The shape of protibiae (character 11) varies between explanate, distal margin width exceeding 1/3 of protibial length, and not explanate, distal margin width equal to 1/4 of protibial length.

**Distribution.** With the distributional data published (Kulzer 1958; Peña 1966; Ferrú and Elgueta 2011; Flores and Pizarro-Araya 2012) and from specimens deposited in the examined collections we made approximate maps of the current geographic distribution for each subgenus. New records are reported for some subgenera and enlargement of their distribution. As a result of recent expeditions (Alfaro et al. 2009; Flores et al. 2011) we recorded the subgenera present on Pacific islands and Peninsula Valdés in the Atlantic Ocean. For distribution of the species we used the biogeographic classification by Morrone (2006).

**Estimation of the diversity of each subgenus.** Based on the types of known species (deposited at FMNH, MACN, MLPA, MNHUB, MNHN, MNNC, and NHMB) and the keys of Kulzer (1958), all specimens available in collections were determined and we identified specimens belonging to species to be described. Other unnamed species were found in collecting trips made for our projects in IADIZA and LEULS since 2001 until now. A list of the unnamed species by subgenus was made with these records and the diversity of each subgenus and of the whole genus was estimated, including the species to be described.

**Species list.** Based on the last revision of the genus (Kulzer 1958) and on most recent studies of types and new synonymies (Flores 2007, 2009; Flores and Pizarro-Araya 2010, 2012; Flores et al. 2011), we made a list of species included for each subgenus. Some species were described after Kulzer’s revision (Kaszab 1964, 1969; Molinari 1969; Marcuzzi 1977, 2001) or rediscovered (Flores 2007), so we assigned these species to some subgenus according to the diagnostic characters presented here.

## Results

### Genus Praocis

Eschscholtz, 1829

http://species-id.net/wiki/Praocis

#### Generic characteristics.

The species of *Praocis* can be recognized by having maxillary palps with last segment axe-shaped (apex twice as wide as base), antennomere 3 shorter than 4 + 5 combined, pronotum with anterior margin concave, width of posterior margin exceeding width of anterior margin, single lateral margin slender, expanded, remote from disc, and anterior angles rounded; mesosternum inclined forward, separated from prosternum; elytron with punctuate surface; apterous.

### (1) Subgenus Praocis (Praocis)

Eschscholtz, 1829

Figs 2 , 15–16

#### Type species.

*Praocis rufipes* Eschscholtz, 1829, subsequent designation by Guérin-Méneville (1834: 8-9).

#### Diagnosis.

Clypeus with anterior margin extending beyond to lateral expansion of frons, width of anterior margin not exceeding half the interocular width, clypeal suture as a vertical groove, not covered by frons, clypeus and frons at same level; antennomere 10 wider than long, antennomere 9 longer than antennomere 10, antennomere 11 longer than antennomere 10; apical tomentose sensory patches on antennomere 10 in a dorsally continuous semicircle, on antennomere 11 on distal half; prosternum with a narrow edge on anterior margin; lateral margin of elytron well defined; ventral surface of profemora with a row of setae on anterior edge; protibiae explanate.

#### Distribution.

Species of *Praocis* s. str. are endemic to central and southern Chile and occur from 26°South (Quebrada el León, Atacama Region) to 42°South (Carelmapu, Los Lagos Region) in the biogeographic provinces of Atacama, Coquimbo, Santiago, Maule and Valdivian Forest (Morrone 2006) (Fig. 15).

#### New records.

We present new records for some Pacific islands. We recorded *Praocis (Praocis) spinolai* Gay & Solier for Damas (29°13'S, 71°31'W), Gaviota (29°15'S, 71°28'W) and Choros (29°15'S, 71°32'W) islands (Alfaro et al. 2009), *Praocis (Praocis) subaenea* Erichson and *Praocis (Praocis) curta* Solier for Chañaral Island (29°02'S, 71°36'W) (pers. obs), and *Praocis (Praocis) costata* Gay & Solier was recorded for Mocha Island (38°23'S, 73°52'W) (Flores and Pizarro-Araya 2012).

#### Diversity.

This subgenus contains 18 species of which 2 species were recently described (Flores and Pizarro-Araya 2012), increasing 13 percent the number of species (Fig. 33).

#### Habitat.

The distribution range of the subgenus extends from sea level to an altitude of ~1300 m. Most species are distributed between the Huasco coastal desert and the coastal shrub steppe (Gajardo 1994), with 4 and 10 species each, and are ecologically related to shrub and herbaceous vegetation (perennial and annual) characteristic of the Chilean Coastal Desert (CCD), in sandy soils or clayey, poorly-permeable soils (Flores and Pizarro-Araya 2012; Cortés-Contreras et al. 2013; collection data FMNH, IADIZA, LEULS, and pers. obs.). One species (*Praocis (Praocis) costata* Solier) inhabits deciduous woodlands of *Nothofagus* spp. (Gajardo 1994) in the Valdivian Forest biogeographic province (Morrone 2006) (Fig. 16).

#### Species included.

*Praocis rufipes* Eschscholtz, 1829 (= *Sternodes mannerheimi* Fischer, 1844, male, synonymy by Motschulsky 1845) (= *Praocis interrupta* Solier, 1851, synonymy by Kulzer 1958); *Praocis costata* Gay & Solier in Solier, 1840 (= *Praocis ciliata* Germain, 1855, synonymy by Kulzer 1958); *Praocis sanguinolenta* Gay & Solier in Solier, 1840 (= *Praocis audouini* Solier, 1840, synonymy by Flores 2007); *Praocis quadrisulcata* Germain, 1855; *Praocis curta* Solier, 1840 (= *Praocis nigroaenea* Solier, 1840, synonymy by Kulzer 1958) (= *Praocis rugipennis* Germain, 1855, synonymy by Kulzer 1958); *Praocis hirtella* Kulzer, 1958a; *Praocis sulcata* Eschscholtz, 1829 (= *Sternodes mannerheimi* Fischer, 1844, female, synonymy by Motschulsky 1845) (= *Praocis rotundata* Laporte, 1840, synonymy by Flores and Pizarro-Araya 2010); *Praocis subsulcata* Gay & Solier in Solier, 1840; *Praocis spinolai* Gay & Solier in Solier, 1840; *Praocis aenea* Gay & Solier in Solier, 1840; *Praocis parva* Gay & Solier in Solier, 1840; *Praocis tibialis* Gay & Solier in Solier, 1840 (= *Praocis rufitarsis* Gay & Solier in Solier, 1840, synonymy by Flores 2007) (= *Praocis aenipennis* Germain, 1855, synonymy by Kulzer 1958); *Praocis subaenea* Erichson, 1834 (= *Praocis submetallica* Guérin-Méneville, 1834, synonymy by Flores 2007) (= *Praocis laevicosta* Curtis, 1845, synonymy by Kulzer 1958); *Praocis marginata* Germain, 1855; *Praocis elliptica* Philippi & Philippi, 1864 (= *Praocis angustata* Philippi & Philippi, 1864, synonymy by Kulzer 1958); *Praocis bicentenario* Flores & Pizarro-Araya, 2012; *Praocis medvedevi* Flores & Pizarro-Araya, 2012; *Praocis bicostata* Philippi & Philippi, 1864 (type lost, assigned to (*Praocis*) by Kulzer 1958).

### (2) Subgenus Praocis (Mesopraocis)

Flores & Pizarro-Araya subgen. n.

http://zoobank.org/C6C1EBD7-2CD9-4698-8D8A-A0823A43B03A

Figs 7 , 17–18

#### Type species.

*Praocis calderana* Kulzer, 1958, present designation.

#### Diagnosis.

Clypeus with anterior margin extending beyond to lateral expansion of frons, width of anterior margin not exceeding half the interocular width, clypeal suture as a vertical groove, not covered by frons, clypeus and frons at same level; antennomere 10 wider than long, antennomere 9 of equal length to 10, antennomere 11 of equal length to 10; apical tomentose sensory patches on antennomere 10 in two areas subequal in size, on antennomere 11 on distal third; prosternum with a narrow edge on anterior margin; lateral margin of elytron not defined; ventral surface of profemora with a row of setae on anterior edge, protibiae very explanate.

#### Distribution.

Species of *Praocis (Mesopraocis)* are endemic to northern Chile and occur from 25°South (Paposo, Antofagasta Region) to 31°South (Caleta Limarí, Coquimbo Region) in the biogeographic provinces of Atacama and Coquimbo (Morrone 2006) (Fig. 17).

#### New records.

We present new records for some Pacific islands. We recorded the species *Praocis (Mesopraocis) pilula* Laporte and *Praocis (Mesopraocis) flava* Kulzer for Damas (29°13'S, 71°31'W) and Gaviota Islands (29°15'S, 71°28'W) (Alfaro et al. 2009).

#### Diversity.

This subgenus contains 4 species (Kulzer 1958) plus 1 species to be described, 5 species in total, with a 25 percent increase in the number of species (Fig. 33).

#### Habitat.

The distribution range of the subgenus extends from sea level to an altitude of ~1325 m. All *Mesopraocis* species are associated with coastal dunes stabilized with vegetation or paleodunes in the transitional coastal desert of Chile and have nocturnal habits, remaining during the day under stones or plants (Cortés-Contreras et al. 2013, collection data FMNH, IADIZA, LEULS, MNNC, and pers. obs.) (Fig. 18).

#### Species included.

*Praocis pilula* Laporte, 1840 (= *Coelus hirticollis* Solier, 1840, synonymy by Lacordaire 1859); *Praocis calderana* Kulzer, 1958; *Praocis flava* Kulzer, 1958; *Praocis nitens* Kulzer, 1959.

**Figures 7–10.**
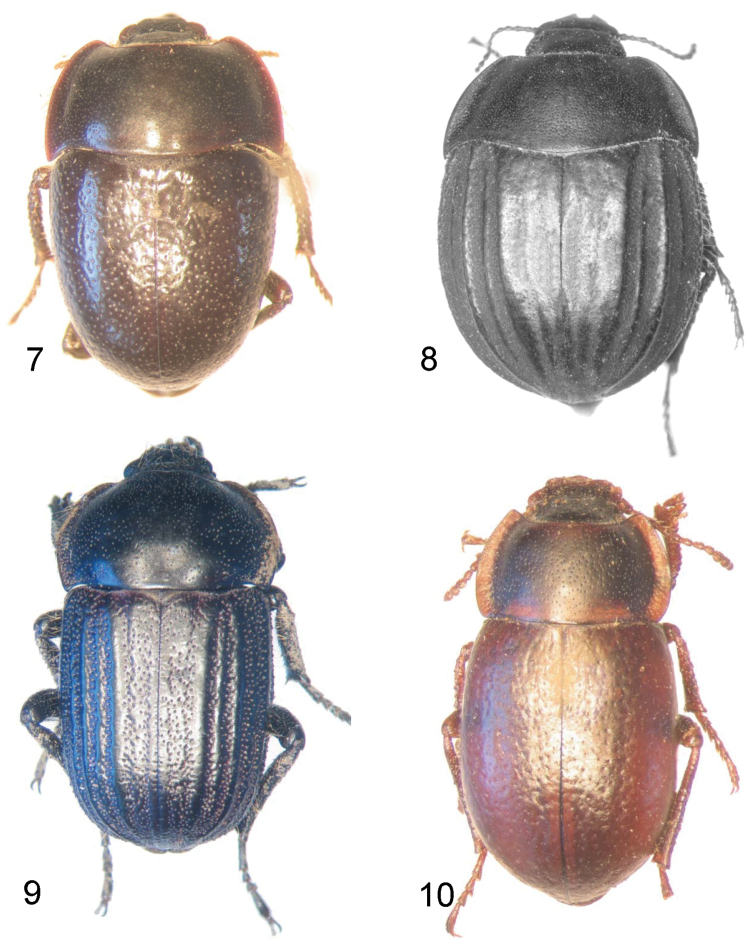
Dorsal view of *Praocis* species. **7** *Praocis (Mesopraocis) calderana*, paratype **8** *Praocis (Postpraocis) pentachorda*, lectotype (previously published in Flores 2009, Zootaxa 1985: Fig. 3; copyright Magnolia Press, reproduced with permission) **9** *Praocis (Anthrasomus) chevrolatii nigra* **10** *Praocis (Filotarsus) peltata*.

### (3) Subgenus Praocis (Postpraocis)

Flores & Pizarro-Araya subgen. n.

http://zoobank.org/2EA923F4-48C6-4DA4-A0C7-A7E3B6714881

Figs 8 , 19–20

#### Type species.

*Praocis pentachorda* Burmeister, 1875, present designation.

#### Diagnosis.

Clypeus with anterior margin extending beyond to lateral expansion of frons, width of anterior margin not exceeding half the interocular width, clypeal suture as a vertical groove, not covered by frons, clypeus and frons at same level; antennomere 10 wider than long, antennomere 9 longer than antennomere 10, antennomere 11 longer than antennomere 10; apical tomentose sensory patches on antennomere 10 in two areas subequal in size, on antennomere 11 on distal half; prosternum with a narrow edge on anterior margin; lateral margin of elytron well defined; ventral surface of profemora without a row of setae on anterior edge, protibiae not explanate.

#### Distribution.

Species of *Praocis (Postpraocis)* inhabit central and northern Chile and western and northern Argentina. They occur from 19°South (Termas de Enquelga, Colchane, Tarapacá Region, Chile) to 34°South in Chile (Rancagua) and 33°South in Argentina (Mendoza) in the biogeographic provinces of Atacama, Coquimbo, Santiago, Puna, Prepuna and Monte (Morrone 2006) (Fig. 19).

#### New records.

We present new records of *Praocis (Postpraocis) pentachorda* Burmeister for the Region Tarapacá of Chile and southern Bolivia and of *Praocis (Postpraocis) curtisi* Solier for the Pacific islands Damas (29°13'S, 71°31'W), Gaviota (29°15'S, 71°28'W) and Choros (29°15'S, 71°32'W) (Alfaro et al. 2009; Ferrú and Elgueta 2011; collection data).

#### Diversity.

This subgenus contains 7 species/subspecies (Kulzer 1958; Flores 2007, 2009) plus 3 species to be described, 10 species in total, with a 43 percent increase in the number of species (Fig. 33).

#### Habitat.

Species of *Praocis (Postpraocis)* have diurnal habits, remaining during the night under stones or plants. In central Chile they can be observed walking on coastal plains or in sandy places lying from sea level to an altitude of ~1300 m. In Argentina, northern Chile and Bolivia, they occur from 1600 m in high altitudinal valleys associated with the Andes mountain range to an altitude of 4200 m in the high Puna plateau, in sandy soils or clayey, poorly permeable soils (Ferrú and Elgueta 2011; Cortés-Contreras et al. 2013; collection data FMNH, IADIZA, LEULS, and pers. obs.) (Fig. 20).

#### Species included.

*Praocis curtisii* Solier, 1851; *Praocis costatula* Gay & Solier in Solier, 1840 (= *Praocis angulifera* Philippi & Philippi, 1864, synonymy by Kulzer 1958); *Praocis pubescens* Philippi & Philippi, 1864; *Praocis pentachorda* Burmeister, 1875 (= *Praocis larraini* Marcuzzi, 2001, synonymy by Flores 2009); *Praocis pentachorda minor* Kulzer, 1958; *Praocis aenescens* Kulzer, 1958; *Praocis concinna* Burmeister, 1875.

### (4) Subgenus Praocis (Anthrasomus)

Guérin-Méneville, 1834

Figs 9 , 21–22

#### Type species.

*Anthrasomus chevrolatii* Guérin-Méneville, 1834, monotypy.

#### Diagnosis.

Clypeus with anterior margin extending beyond to lateral expansion of frons, width of anterior margin not exceeding half the interocular width, clypeal suture as a horizontal groove covered by frons, clypeus lower than frons; antennomere 10 wider than long, antennomere 9 longer than antennomere 10, antennomere 11 longer than antennomere 10; apical tomentose sensory patches on antennomere 10 in two areas subequal in size, on antennomere 11 on distal half; prosternum with a narrow edge on anterior margin; lateral margin of elytron not defined; ventral surface of profemora without a row of setae on anterior edge, protibiae not explanate.

#### Distribution.

Species of *Praocis (Anthrasomus)* inhabit central Chile and occur from 28°South (Freirina, Atacama Region) to 33°South (San Fernando, Libertador General Bernardo O’Higgins Region) in the biogeographic provinces of Atacama, Coquimbo, and Santiago (Morrone 2006) (Fig. 21).

#### Diversity.

This subgenus contains 5 species/subspecies (Kulzer 1958; Flores 2007) plus 1 species to be described, 6 species in total, with a 20 percent increase in the number of species (Fig. 33).

#### Habitat.

Species of *Praocis (Anthrasomus)* have nocturnal habits, remaining during the day under stones or plants in coastal plains, gullies, and transverse valleys in semiarid Chile. They occur from sea level to an altitude of 2800 m, in stony-clayey, poorly permeable soils (collection data FMNH, IADIZA, LEULS, and pers. obs.) (Fig. 22).

#### Species included.

*Praocis chevrolatii* Guérin-Méneville, 1834 (= *Praocis gayi* Solier, 1840, synonymy by Kulzer 1958) (= *Praocis hispidula* Philippi & Philippi, 1864, synonymy by Kulzer 1958) (= *Praocis laticollis* Philippi & Philippi, 1864, synonymy by Kulzer 1958); *Praocis chevrolatii subcostata* Gay & Solier in Solier, 1840 (= *Praocis chevrolatii coquimboana* Kaszab, 1969, synonymy by Flores 2007); *Praocis chevrolatii nigra* Kulzer, 1958; *Praocis hirtuosa* Gay & Solier in Solier, 1840 (= *Praocis pubens* Philippi & Philippi, 1864, synonymy by Kulzer 1958); *Praocis nuda* Kulzer, 1958.

### (5) Subgenus Praocis (Filotarsus)

Gay & Solier in Solier, 1840

Figs 10 , 23–24

#### Type species.

*Filotarsus tenuicornis* Gay & Solier in Solier, 1840, monotypy and original designation by Solier (1840: 241).

#### Diagnosis.

Clypeus with anterior margin extending beyond to lateral expansion of frons or at same level as lateral expansion of frons, width of anterior margin not exceeding half the interocular width or width of anterior margin same as interocular width, clypeal suture as a vertical groove, not covered by frons, clypeus and frons at same level or clypeal suture as a horizontal groove not covered by frons, clypeus lower than frons; antennomere 9 longer than antennomere 10, antennomere 11 longer than antennomere 10; apical tomentose sensory patches on antennomere 10 in a dorsally continuous semicircle, on antennomere 11 on distal half; prosternum with a narrow edge on anterior margin; lateral margin of elytron not defined; ventral surface of profemora without a row of setae on anterior edge, protibiae explanate.

#### Distribution.

Species of *Praocis (Filotarsus)* inhabit central and northern Chile, western and northern Argentina, estern Bolivia and southern Peru. They occur from 12°South (Cuzco, Peru) to 39°South (Neuquén, Argentina) in the biogeographic provinces of Puna, Atacama, Coquimbo, Santiago, Prepuna, Monte, and Central Patagonia (Morrone 2006) (Fig. 23).

#### Diversity.

This subgenus contains 14 species (Kulzer 1958; Flores 2009) plus 6 species to be described, 20 species in total, with a 43 percent increase in the number of species (Fig. 33).

#### Habitat.

Species of *Praocis (Filotarsus)* have nocturnal habits, remaining during the day under stones or plants. In central Chile they can be observed in gullies and Coastal and Andean mountain ranges from 400 m to an altitude of 2500 m. In Argentina, Bolivia, northern Chile and Peru they occur from 1600 m in high altitudinal valleys associated with the Andes mountain range to an altitude of 5200 m in the high Puna plateau, in clayey, poorly permeable soils (Ferrú and Elgueta 2011; collection data FMNH, IADIZA, LEULS, and pers. obs.) (Fig. 24).

#### Species included.

*Praocis tenuicornis* Gay & Solier in Solier, 1840; *Praocis castanea* Germain, 1855; *Praocis rufilabris* Gay & Solier in Solier, 1840; *Praocis uretai* Kulzer, 1958 (= *Praocis freyi* Marcuzzi, 1977, synonymy by Flores 2009); *Praocis reedi* Kulzer, 1958; *Praocis oblonga* Solier, 1851; *Praocis peltata* Erichson, 1834; *Praocis forsteri* Kulzer, 1958; *Praocis obesa* Kulzer, 1958; *Praocis titschacki* Kulzer, 1958; *Praocis brevicornis* Kulzer, 1958; *Praocis weyrauchi* Kulzer, 1958; *Praocis peruana* Fairmaire, 1902; *Praocis grossa* Kulzer, 1958.

### (6) Subgenus Praocis (Hemipraocis)

Flores & Pizarro-Araya subgen. n.

http://zoobank.org/1EF65CCD-D4C2-4B9F-BB5B-7BB98170599D

Figs 11 , 25–26

#### Type species.

*Praocis sellata* Berg, 1889, present designation.

#### Diagnosis.

Clypeus with anterior margin extending beyond to lateral expansion of frons, width of anterior margin not exceeding half the interocular width, clypeal suture as a horizontal groove not covered by frons, clypeus lower than frons; antennomere 9 longer than antennomere 10, antennomere 11 longer than antennomere 10; apical tomentose sensory patches on antennomere 10 in two areas subequal in size, on antennomere 11 on distal half; prosternum without a narrow edge on anterior margin; lateral margin of elytron well defined; ventral surface of profemora without a row of setae on anterior edge, protibiae explanate.

#### Distribution.

The species of *Praocis (Hemipraocis)* occur from central Argentina (southern Mendoza 36°S and coastal Buenos Aires 36°S), to southern Argentina and Chile (northern Magellan Strait 52°S), in the biogeographic provinces of Patagonia, Monte and Pampa (Morrone 2006) (Fig. 25).

#### New records.

We present a new record for the Peninsula Valdés in Argentina (Flores et al. 2011).

#### Diversity.

This subgenus contains 8 species/subspecies (Kulzer 1958; Flores 2007, 2009; Flores et al. 2011) of which 2 subspecies were recently described (Flores et al. 2011), plus 8 species to be described, 16 species in total, with a 167 percent increase in the species number (Fig. 33).

#### Habitat.

Species of *Praocis (Hemipraocis)* have diurnal and crepuscular habits, hiding during the night under shrubs, stones or buried in sand. They inhabit the Patagonian steppes and coastal Pampa from sea level to an altitude of 1700 m, in sandy soils or clayey, poorly permeable soils (Flores et al. 2011; collection data FMNH, IADIZA, and pers. obs.) (Fig. 26).

#### Species included.

*Praocis sellata* Berg, 1889; *Praocis sellata bergi* Kulzer, 1958; *Praocis sellata bruchi* Kulzer, 1958 (= *Praocis sellata topali* Kaszab, 1964, synonymy by Flores et al. 2011); *Praocis sellata peninsularis* Flores & Carrara, 2011 (in Flores et al. 2011); *Praocis sellata granulipennis* Flores & Carrara, 2011 (in Flores et al. 2011); *Praocis fimbriata* Burmeister, 1875; *Praocis striolicollis* Fairmaire, 1883a (= *Praocis denseciliata* Fairmaire, 1883b, synonymy by Flores 2007) (= *Praocis silvestrii* Marcuzzi, 2001, synonymy by Flores 2009); *Praocis inermis* Burmeister, 1875 (= *Praocis compacta* Fairmaire, 1883b, synonymy by Flores 2007).

**Figures 11–14.**
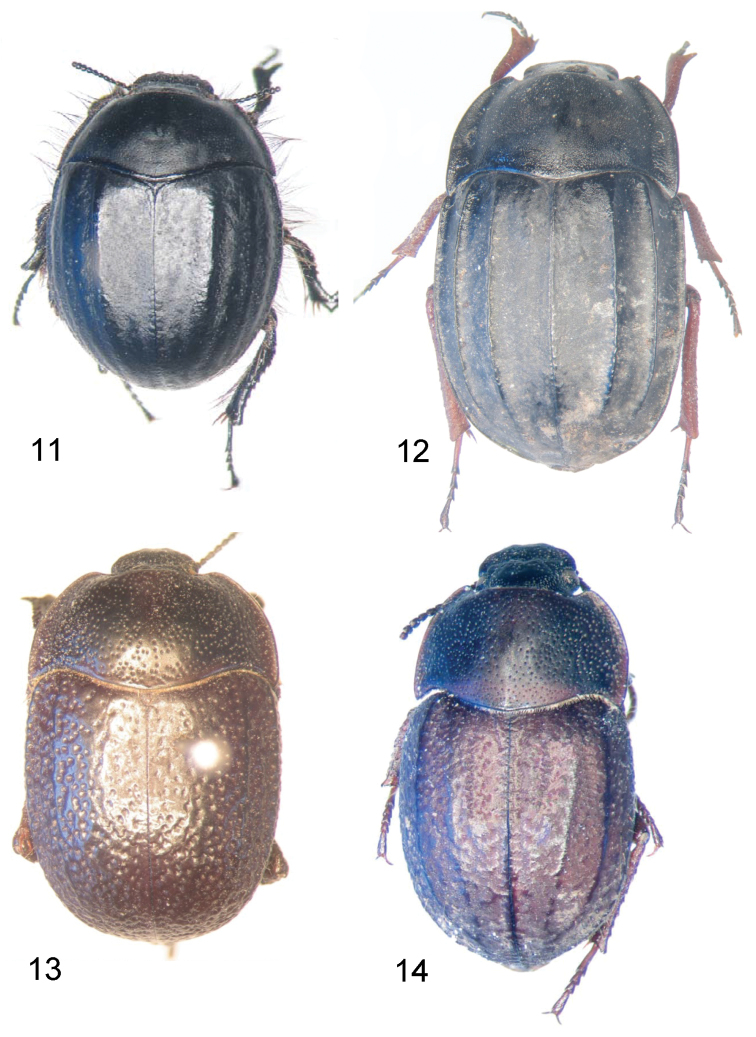
Dorsal view of *Praocis* species. **11** *Praocis (Hemipraocis) sellata peninsularis*, holotype (reproduced from Flores et al. 2011) **12** *Praocis (Praonoda) bicarinata* **13** *Praocis (Orthogonoderes) ecostata*, holotype **14** *Praocis (Praocida) montana*, holotype (previously published in Flores 2009, Zootaxa 1985: Fig. 9; copyright Magnolia Press, reproduced with permission).

### (7) Subgenus Praocis (Praonoda)

Flores & Pizarro-Araya subgen. n.

http://zoobank.org/5D327D83-AAE6-4E40-B7BC-B338ADE4CA09

Figs 12 , 27–28

#### Type species.

*Praocis bicarinata* Burmeister, 1875, present designation.

#### Diagnosis.

Clypeus with anterior margin extending beyond to lateral expansion of frons, width of anterior margin not exceeding half the interocular width, clypeal suture as a horizontal groove not covered by frons, clypeus lower than frons; antennomere 9 longer than antennomere 10, antennomere 11 longer than antennomere 10; apical tomentose sensory patches on antennomere 10 in two areas subequal in size, on antennomere 11 on distal half; prosternum without a narrow edge on anterior margin; lateral margin of elytron well defined; ventral surface of profemora without a row of setae on anterior edge, protibiae explanate.

#### Distribution.

The species of *Praocis (Praonoda)* occur from Neuquén and Rio Negro provinces in Argentina (40°S) to northern Tierra del Fuego Island (52°30'S) with the species *Praocis (Praonoda) bicarinata* as the unique species of *Praocis* inhabiting Tierra del Fuego. They inhabit the biogeographic provinces of Patagonia and Monte (Morrone 2006) (Fig. 27).

#### Diversity.

This subgenus contains 2 species (Kulzer 1958) plus 2 species to be described, 4 species in total, with a 100 percent increase in the number of species (Fig. 33).

#### Habitat.

Species of *Praocis (Praonoda)* have diurnal and crepuscular habits, hiding during the night under shrubs or stones. They inhabit the Patagonian steppes from sea level to an altitude of 1250 m, in sandy soils or clayey, poorly permeable soils (collection data FMNH, IADIZA and pers. obs.) (Fig. 28).

#### Species included.

*Praocis bicarinata* Burmeister, 1875 (= *Praocis silphomorpha* Fairmaire, 1883a, synonymy by Berg 1884); *Praocis molinari* Kulzer, 1958.

### (8) Subgenus Praocis (Orthogonoderes)

Gay & Solier in Solier, 1840

Figs 13 , 29–30

Aulacus Gray, 1832: 783. Type species: *Aulacus chilensis* Gray, 1832, monotypy. Synonymy by Kulzer (1958).
Eurygona Laporte, 1840: 187. Type species: *Aulacus chilensis* Gray, 1832, monotypy. Synonymy by Kulzer (1958).

#### Type species.

*Praocis subreticulata* Gay & Solier in Solier, 1840, present designation.

#### Diagnosis.

Clypeus with anterior margin extending beyond to lateral expansion of frons, width of anterior margin not exceeding half the interocular width, clypeal suture as a horizontal groove covered by frons, clypeus lower than frons; antennomere 9 longer than antennomere 10, antennomere 11 shorter than antennomere 10; apical tomentose sensory patches on antennomere 10 in two areas subequal in size, on antennomere 11 on distal third; prosternum without a narrow edge on anterior margin; lateral margin of elytron well defined; ventral surface of profemora without a row of setae on anterior edge, protibiae explanate.

#### Distribution.

Species of *Praocis (Orthogonoderes)* inhabit central and northern Chile, western and northern Argentina, western Bolivia and southern Peru. They occur from 12°South (Cuzco, Peru) to 38°South in Chile (Nahuelbuta) and 39°South in Argentina (Neuquén) in the biogeographic provinces of Puna, Atacama, Coquimbo, Santiago, Maule, Prepuna, Monte, and Central Patagonia (Morrone 2006). One species (*Praocis (Orthogonoderes) insularis* Kulzer) has been recorded in the Guacolda island in the Pacific Ocean (28°S) (Kulzer 1958; Peña 1966) (Fig. 29).

#### New records.

We present a new record of *Praocis argentina* Kulzer for the Atlantic coast in Argentina, the isthmus of Peninsula Valdés, 42°30'S.

#### Diversity.

This subgenus contains 23 species (Kulzer 1958; Flores 2007, 2009) plus 10 species to be described, 33 species in total, with a 43 percent increase in the number of species (Fig. 33).

#### Habitat.

Species of *Praocis (Orthogonoderes)* have diurnal and crepuscular habits, hiding during the night under shrubs or stones. In central Chile they can be observed in coastal dunes stabilized with vegetation or paleodunes, gullies, coastal plains, transverse valleys and Coastal and Andean mountain ranges from sea level to an altitude of 2700 m. In Argentina, Bolivia, Peru, and northern Chile, they occur from 1600 m high altitudinal valleys associated with the Andes mountain range to an altitude of 4200 m in the high Puna plateau, in sandy soils or in clayey, poorly permeable soils (Cortés-Contreras et al. 2013; collection data FMNH, IADIZA, LEULS, and pers. obs). The only species inhabiting Patagonian steppes, *Praocis (Orthogonoderes) argentina*, is recorded from 1700 m in southern Mendoza to sea level on the Atlantic coast in Argentina (collection data IADIZA, LEULS, and pers. obs.). *Orthogonoderes* is the only subgenus inhabiting both the Pacific and Atlantic coasts of South America (Fig. 30).

#### Species included.

*Praocis cribrata* Gay & Solier in Solier, 1840; *Praocis adspersa* Germain, 1855; *Praocis depressicollis* Germain, 1855; *Praocis ecostata* Kulzer, 1958; *Praocis subreticulata* Gay & Solier in Solier, 1840; *Praocis dentipes* Germain, 1855; *Praocis pleuroptera* Gay & Solier in Solier, 1840 (= *Praocis convexa* Germain, 1855, synonymy by Flores 2007); *Praocis plicicollis* Germain, 1855; *Praocis laevicollis* Philippi & Philippi, 1864 (= *Praocis nitidicollis* Philippi & Philippi, 1864, synonymy by Kulzer 1958); *Praocis ebenina* Germain, 1855; *Praocis picipes* Germain, 1855 (= *Praocis consobrina* Philippi & Philippi, 1864, synonymy by Kulzer 1958) (= *Praocis rotundicollis* Philippi & Philippi, 1864, synonymy by Kulzer 1958); *Praocis costipennis* Solier, 1851; *Praocis rugata* Gay & Solier in Solier, 1840; *Praocis punctata* Gay & Solier in Solier, 1840; *Praocis rotundata* Lacordaire, 1830 (= *Praocis soror* Kulzer, 1958, synonymy by Flores and Pizarro-Araya 2010); *Praocis variolosa* Erichson, 1834; *Praocis variolosa laxepunctata* Kulzer, 1958; *Praocis penai* Kulzer, 1958 (incorrect original spelling: *peñai*, Article 32.5 ICZN 1999); *Praocis chilensis* (Gray, 1832); *Praocis insularis* Kulzer, 1958; *Praocis tibiella* Kulzer, 1958; *Praocis argentina* Kulzer, 1962; *Praocis magnoi* Molinari, 1969.

### (9) Subgenus Praocis (Praocida)

Flores & Pizarro-Araya subgen. n.

http://zoobank.org/4B6EC138-1D93-4567-BB66-E3A6B1FC4593

Figs 14 , 31–32

#### Type species.

*Praocis zischkai* Kulzer, 1958, present designation.

#### Diagnosis.

Clypeus with anterior margin extending beyond to lateral expansion of frons, width of anterior margin not exceeding half the interocular width, clypeal suture as a horizontal groove not covered by frons, clypeus lower than frons; antennomere 9 longer than antennomere 10, antennomere 11 longer than antennomere 10; apical tomentose sensory patches on antennomere 10 in two areas subequal in size, on antennomere 11 on distal third; prosternum without a narrow edge on anterior margin; lateral margin of elytron well defined; ventral surface of profemora without a row of setae on anterior edge, protibiae explanate.

#### Distribution.

Species of *Praocis (Praocida)* inhabit southern Peru, central and southern Bolivia and northern Argentina. They occur from 12°South (Cuzco, Peru) to 31°South in Cordoba (northern Argentina), in the biogeographic provinces of Puna, Chaco, and Pampa (Morrone 2006) (Fig. 31). *Praocida* is the only subgenus of *Praocis* inhabiting the biogeographic province of Chaco.

#### New records.

We present a new record of *Praocis (Praocida) teniucosta* Kulzer for the mountains in South Buenos Aires province (38°S).

#### Diversity.

This subgenus contains 4 species (Kulzer 1958; Flores 2009) plus 3 species to be described, 7 species in total, with a 75 percent increase in number of species (Fig. 33).

#### Habitat.

Species of *Praocis (Praocida)* have nocturnal habits, hiding during the day under shrubs, stones or logs in clayey, poorly permeable soils. They occur from 1200 m in the Chacoan forest to an altitude of 4000 m in Puna (collection data FMNH, IADIZA and pers. obs.) (Fig. 32).

#### Species included.

*Praocis tenuicosta* Kulzer, 1958; *Praocis zischkai* Kulzer, 1958; *Praocis kuscheli* Kulzer, 1958; *Praocis montana* Kulzer, 1958 (= *Praocis baloghi* Marcuzzi, 2001, synonymy by Flores 2009).

#### Species of *Praocis* incertae sedis.

*Praocis pentagona* Lacordaire, 1830; *Praocis squalida* Lacordaire, 1830; *Praocis silphoides* Lacordaire, 1830; *Praocis spinipes* Laporte, 1840; *Praocis hirticollis* Laporte, 1840. Type material belonging to these five species is missing (Kulzer 1958; Flores and Pizarro-Araya 2010) and the original descriptions do not provide information for the subgeneric assignment.

### Key to the subgenera of *Praocis*

**Table:** 

1	Anterior margin of prosternum with a narrow, sharp edge	2
–	Anterior margin of prosternum rounded, smooth, lacking edge	6
2	Lateral margin of elytron well defined by a sharp edge carina-shaped, narrow or broad (Figs 2, 8), dorsal area of elytron well differentiated from pseudopleuron	3
–	Lateral margin of elytron not defined, rounded (Figs 7, 9–10), surface continuous between dorsal area of elytron and pseudopleuron	4
3	Apical tomentose sensory patches on antennomere 10 arranged in a dorsally continuous semicircle (Fig. 3); ventral surface of profemora with a row of setae on anterior edge (Fig. 2)	*Praocis* Eschscholtz
–	Apical tomentose sensory patches on antennomere 10 arranged in two areas subequal in size (Fig. 4); ventral surface of profemora lacking a row of setae on anterior edge (Fig. 8)	*Postpraocis* Flores & Pizarro-Araya
4	Antennae very short, reaching only 1/4 of lateral margin of pronotum; antennomere 9 equal length as antennomere 10; antennomere 11 equal length as antennomere 10; apical tomentose sensory patches on antennomere 11 on distal third (Fig. 4); ventral surface of profemora with a row of setae on anterior edge (Fig. 7)	*Mesopraocis* Flores & Pizarro-Araya
–	Antennae long, reaching or surpassing the midpoint of lateral margin of pronotum; antennomere 9 longer than antennomere 10 (Fig. 3); antennomere 11 longer than antennomere 10 (Fig. 3); apical tomentose sensory patches on antennomere 11 on distal half (Fig. 3); ventral surface of profemora lacking a row of setae on anterior edge	5
5	Apical tomentose sensory patches on antennomere 10 arranged in two areas subequal in size (Fig. 4); dorsal area of elytron with 2 to 5 carinae; protibiae not explanate (Fig. 9)	*Anthrasomus* Guérin-Méneville
–	Apical tomentose sensory patches on antennomere 10 arranged in a dorsally continuous semicircle (Fig. 3); dorsal area of elytron lacking carinae; protibiae explanate (Fig. 10)	*Filotarsus* Gay & Solier
6	Apical tomentose sensory patches on antennomere 11 on distal half (Fig. 3)	7
–	Apical tomentose sensory patches on antennomere 11 on distal third (Fig. 4)	8
7	Body spherical, wide, rounded seen from above; lateral margin of elytra as a wide, prominent edge; lateral margin of pronotum with a row of long, black or golden setae (Fig. 11)	*Hemipraocis* Flores & Pizarro-Araya
–	Body elongate, narrow, subparallel seen from above, lateral margin of elytra as sharp edge carina-shaped; lateral margin of pronotum lacking a row of setae (Fig. 12)	*Praonoda* Flores & Pizarro-Araya
8	Antennomere 11 shorter than antennomere 10; clypeal suture as a horizontal groove covered by frons (Figs 4, 13)	*Orthogonoderes* Gay & Solier
–	Antennomere 11 longer than antennomere 10; clypeal suture as a horizontal groove not covered by frons (Figs 3, 14)	*Praocida* Flores & Pizarro-Araya

**Figures 15–18.**
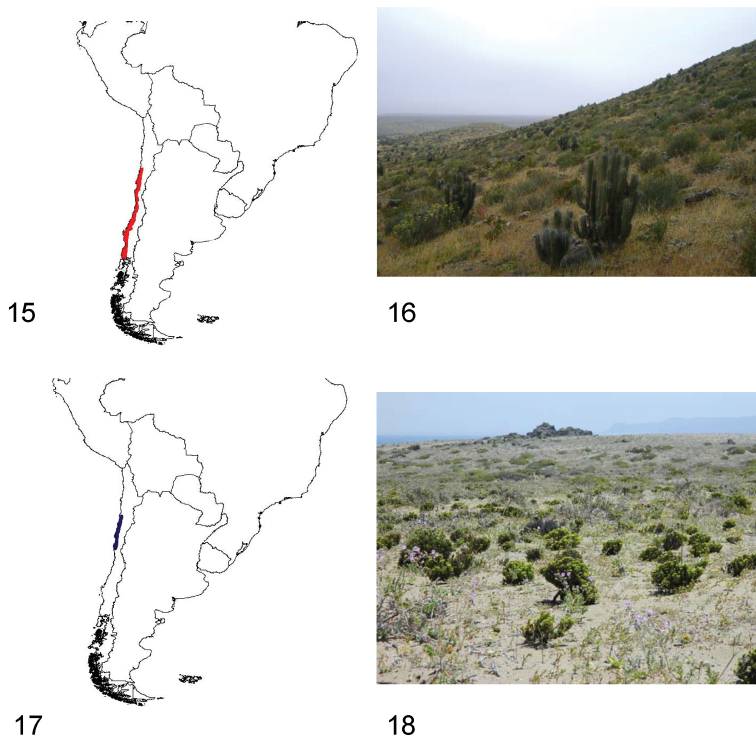
**15** Distribution area of the subgenus *Praocis (Praocis)* **16** Punta de Choros (Coquimbo Region, Chile), habitat of *Praocis (Praocis) spinolai* **17** Distribution area of the subgenus *Praocis (Mesopraocis)* **18** Chañaral de Aceituno, Huasco (Atacama Region, Chile), habitat of *Praocis (Mesopraocis) pilula*.

**Figures 19–22.**
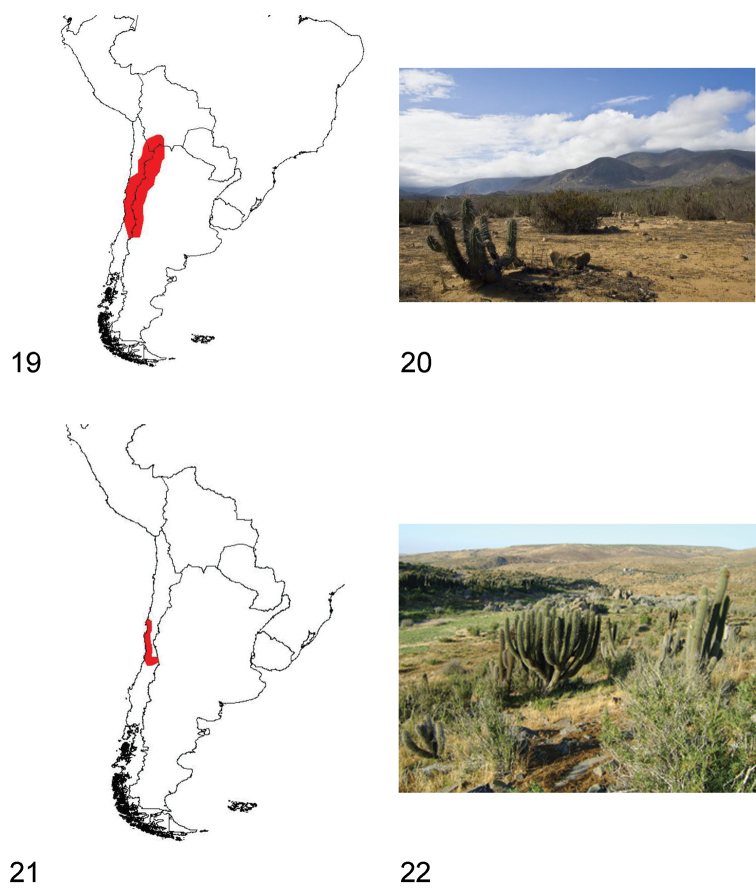
**19** Distribution area of the subgenus *Praocis (Postpraocis)* **20** Totoralillo Norte (Coquimbo Region, Chile), habitat of *Praocis (Postpraocis) curtisii* **21** Distribution area of the subgenus *Praocis (Anthrasomus)* **22** Socos (Coquimbo Region, Chile), habitat of *Praocis (Anthrasomus) chevrolatii subcostata*.

**Figures 23–26.**
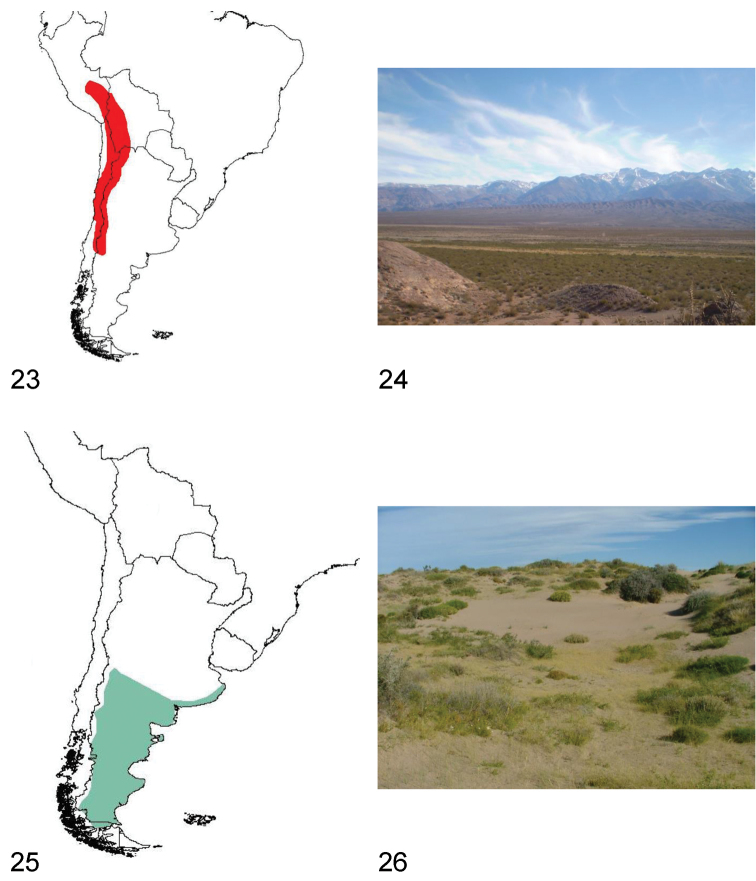
**23** Distribution area of the subgenus *Praocis (Filotarsus)* **24** Uspallata Valley (Mendoza, Argentina), habitat of *Praocis (Filotarsus) oblonga* **25** Distribution area of the subgenus *Praocis (Hemipraocis)* **26** Peninsula Valdés (Chubut, Argentina), habitat of *Praocis (Hemipraocis) sellata peninsularis*.

**Figures 27–30.**
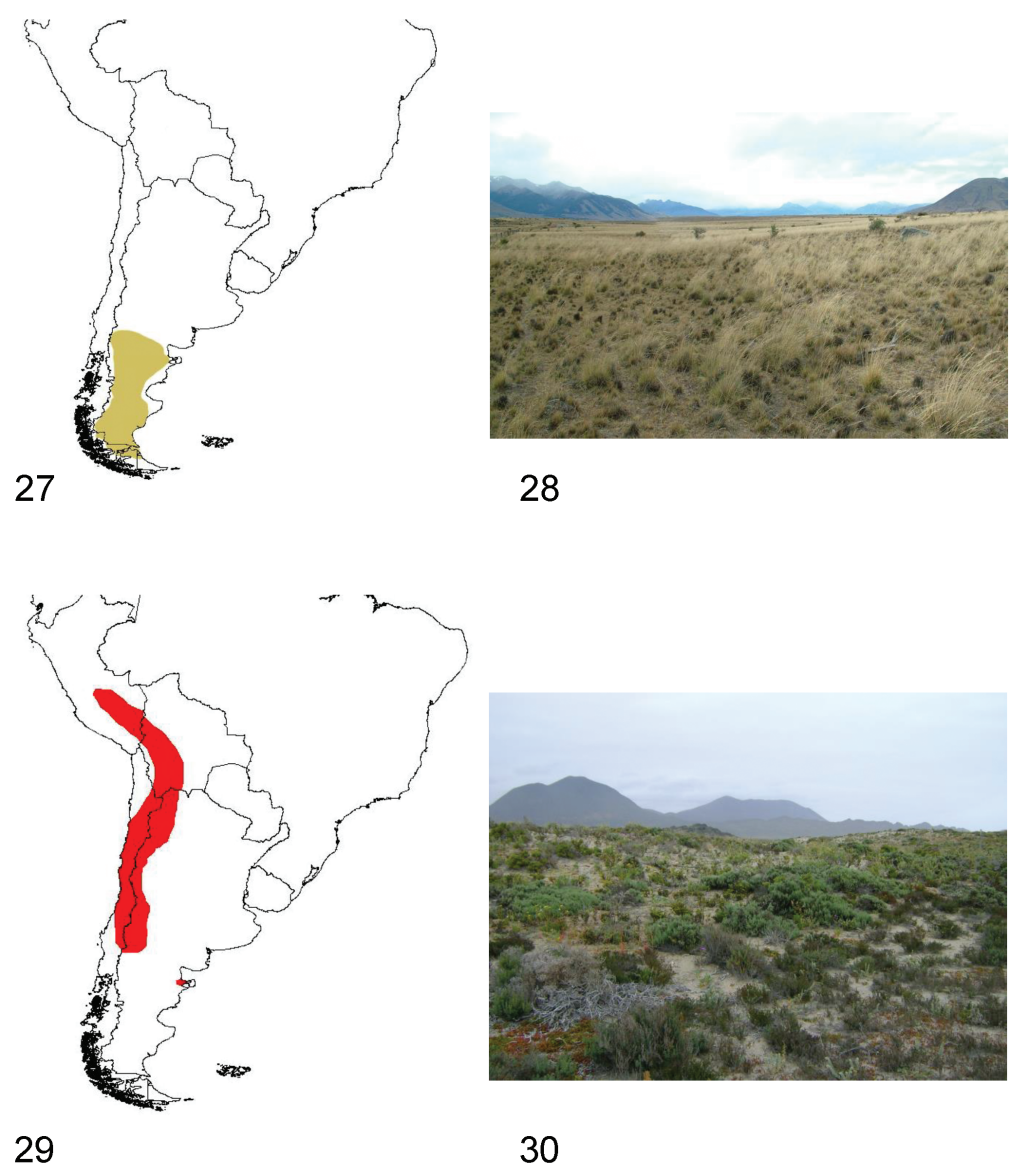
**27** Distribution area of the subgenus *Praocis (Praonoda)* **28** Southern Santa Cruz (Argentina), habitat of *Praocis (Praonoda) bicarinata* **29** Distribution area of the subgenus *Praocis (Orthogonoderes)* **30** Choros Bajos, (Coquimbo Region, Chile), habitat of *Praocis (Orthogonoderes) chilensis*.

**Figures 31–32.**
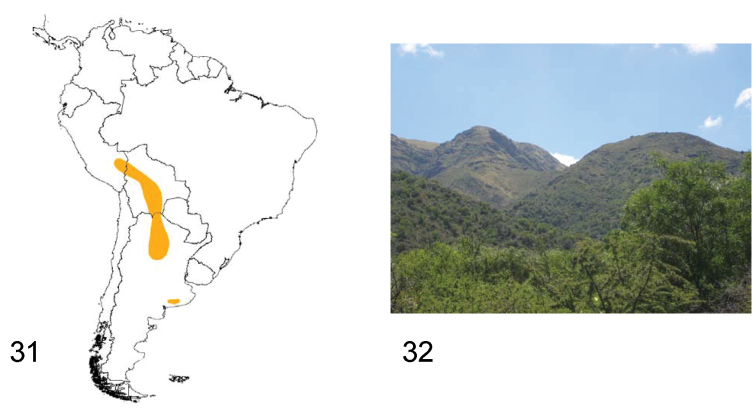
**31** Distribution area of the subgenus *Praocis (Praocida)* **32** Capilla del Monte (Córdoba, Argentina), habitat of *Praocis (Praocida) teniucosta* (Photo by Liliana Arguello).

**Figure 33.**
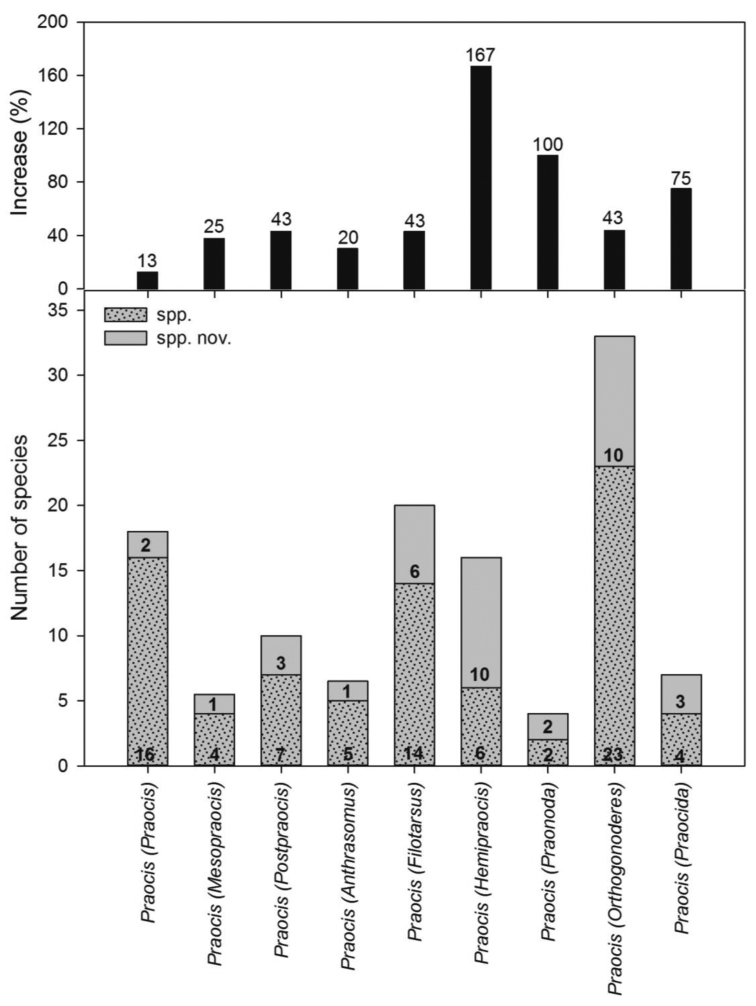
Diversity of the subgenera of *Praocis*. Current number of species (dotted); number of species to be described or recently described (grey) and percentage of increasing of species for each subgenus (black).

## Discussion

### Estimation of the diversity of the whole genus

*Praocis* currently contains 77 species and 8 subspecies (Flores and Pizarro-Araya 2012) arranged in 9 subgenera (Fig. 33). Taking into account the 34 currently undescribed species, the genus will have 119 species/subspecies (Fig. 33), with a 47 percent increase in the number of species in the entire genus. All these undescribed species fall within the present generic concept of *Praocis*. An assessment of the subgeneric characters presented herein among these species to be described show a preliminary affiliation as detailed in Fig. 33. Three species that did not fit in any generic concept of Praociini or subgeneric concept of *Praocis* were recently described in a new genus, *Patagonopraocis* (Flores and Chani-Posse 2005).

### Character states

A table was made with the character states used in the diagnoses (Table 1). This table summarizes the distribution of character states among the subgenera. It can be observed that each subgenus can be defined by a particular combination of these characters, stated in each diagnosis. For the characters here named 1-3, different species of *Filotarsus* present both the states found for each character, which are constant and well defined in all the species of the other subgenera, suggesting that in *Filotarsus* there are at least two groups of species which will be elucidated further by examining all the species of the subgenus and conducting a cladistic analysis of the group.

**Table 1.** Characters studied and distribution of character states among the subgenera of *Praocis*.

Characters	Character states	Subgenera								
		*Praocis* s. str.	*Mesopraocis*	*Postpraocis*	*Anthrasomus*	*Filotarsus*	*Hemipraocis*	*Praonoda*	*Orthogonoderes*	*Praocida*
1) Clypeus, anterior margin	A) extending beyond to lateral expansion of frons	×	×	×	×	×	×	×	×	×
	B) at same level as lateral expansion of frons					×				
2) Clypeus, width of anterior margin	A) not exceeding half the interocular width	×	×	×	×	×	×	×	×	×
	B) same width as interocular distance					×				
3) Clypeal suture	A) as horizontal groove				×	×	×	×	×	×
	B) as vertical groove	×	×	×		×				
4) Antennomere 9	A) longer than antennomere 10	×		×	×	×	×	×	×	×
	B) equal in length to antennomere 10		×							
5) Antennomere 11	A) Longer than antennomere 10	×		×	×	×	×	×		×
	B) shorter than antennomere 10								×	
	C) equal in length to antennomere 10		×							
6) Apical tomentose sensory patches on antennomere 10	A) in two areas subequal in size		×	×	×		×	×	×	×
	B) in a dorsally continuous semicircle	×				×				
7) Apical tomentose sensory patches on antennomere 11	A) on distal third		×						×	×
	B) on distal half	×		×	×	×	×	×		
8) Prosternum	A) with a narrow edge on anterior margin	×	×	×	×	×				
	B) without edge on anterior margin						×	×	×	×
9) Lateral margin of elytron	A) well defined	×		×			×	×	×	×
	B) not defined		×		×	×				
10) Ventral surface of profemora	A) with a row of setae on anterior edge	×	×							
	B) without a row of setae on anterior edge			×	×	×	×	×	×	×
11) Protibiae	A) explanate	×	×			×	×	×	×	×
	B) not explanate			×	×					

Some character states appear as unique for some subgenera such as antennomere 11 of equal length to 10 in *Mesopraocis* and antennomere 11 shorter than antennomere 10 in *Orthogonoderes*. One third of the characters analysed here are from the antennae, suggesting the importance of studying the length and proportion of antennomeres 9, 10 and 11 and the arrangement of the apical tomentose sensory patches on antennomeres 9, 10 and 11. Using these character states, we presented a preliminary identification key for the subgenera of *Praocis*.

## Biogeography

The distribution of the whole genus *Praocis* is related to the arrangement of the Andes mountain range in southern South America. The Andes are the only high mountain chain in the continent, running along the Pacific coast of South America from Venezuela down South to Tierra del Fuego, extending over 8500 km and separating xeric habitats both eastward and westward (Flores and Pizarro-Araya 2006). Among the genera of Pimeliinae, the distributional patterns of the nine subgenera of *Praocis* were analysed in relation to the Andes mountain range. We found three distribution patterns: three endemic subgenera west of the Andes, in central and northern Chile, *Praocis*, *Mesopraocis* and *Anthrasomus* (Figs 15, 17, 21); three endemic subgenera east of the Andes, in Patagonian steppes, Monte, Chaco and eastern Puna (Argentina, Bolivia and Peru), *Hemipraocis*, *Praonoda* and *Praocida* (Figs 25, 27, 31); and three subgenera widely distributed on both sides of the Andes and inhabiting also high altitudes of the Andes, *Postpraocis*, *Filotarsus* and *Orthogonoderes* (Figs 19, 23, 29). Based on these distribution patterns, and despite the current lack of a phylogeny for the genus, we can hypothesize that the ancestor of all *Praocis* species was older than the uplift of these mountains and the distribution of the species of six current subgenera was affected by a vicariant event caused by the uplift of the Andes. This vicariant event, which was analyzed in known phylogenies of tribes and genera of Pimeliinae in South America, left genera and species both east and west of the Andes (Flores and Pizarro-Araya 2006).

## Supplementary Material

XML Treatment for Praocis

XML Treatment for Praocis (Praocis)

XML Treatment for Praocis (Mesopraocis)

XML Treatment for Praocis (Postpraocis)

XML Treatment for Praocis (Anthrasomus)

XML Treatment for Praocis (Filotarsus)

XML Treatment for Praocis (Hemipraocis)

XML Treatment for Praocis (Praonoda)

XML Treatment for Praocis (Orthogonoderes)

XML Treatment for Praocis (Praocida)
